# Circadian Transcription Contributes to Core Period Determination in Drosophila

**DOI:** 10.1371/journal.pbio.0060119

**Published:** 2008-05-20

**Authors:** Sebastian Kadener, Jerome S Menet, Rebecca Schoer, Michael Rosbash

**Affiliations:** 1 Department of Biology, Brandeis University, Waltham, Massachusetts, United States of America; 2 Howard Hughes Medical Institute, Brandeis University, Waltham, Massachusetts, United States of America; 3 National Center for Behavioral Genomics, Brandeis University, Waltham, Massachusetts, United States of America; University of Pennsylvania, United States of America

## Abstract

The Clock–Cycle (CLK–CYC) heterodimer constitutes a key circadian transcription complex in Drosophila. CYC has a DNA-binding domain but lacks an activation domain. Previous experiments also indicate that most of the transcriptional activity of CLK–CYC derives from the glutamine-rich region of its partner CLK. To address the role of transcription in core circadian timekeeping, we have analyzed the effects of a CYC–viral protein 16 (VP16) fusion protein in the Drosophila system. The addition of this potent and well-studied viral transcriptional activator (VP16) to CYC imparts to the CLK–CYC-VP16 complex strongly enhanced transcriptional activity relative to that of CLK–CYC. This increase is manifested in flies expressing CYC-VP16 as well as in S2 cells. These flies also have increased levels of CLK–CYC direct target gene mRNAs as well as a short period, implicating circadian transcription in period determination. A more detailed examination of reporter gene expression in CYC-VP16–expressing flies suggests that the short period is due at least in part to a more rapid transcriptional phase. Importantly, the behavioral effects require a *period* (*per*) promoter and are therefore unlikely to be merely a consequence of generally higher PER levels. This indicates that the CLK–CYC-VP16 behavioral effects are a consequence of increased *per* transcription. All of this also suggests that the timing of transcriptional activation and not the activation itself is the key event responsible for the behavioral effects observed in CYC-VP16-expressing flies. The results taken together indicate that circadian transcription contributes to core circadian function in Drosophila.

## Author Summary

The existence of circadian clocks, which allow organisms to predict daily changes in their environments, have been recognized for centuries, yet only recently has the molecular machinery responsible for their generation been uncovered. The current model in animals posits that interlocked feedback loops of transcription-translation produce these 24-hour rhythms. In fruit flies, the transcription loop contains a key activator complex, composed of the transcription factors Clock and Cycle. This CLK-CYC complex stimulates the synthesis of repressor proteins like Period and Timeless, which repress the activator complex. The synthesis–repression cycle takes precisely 24 hours under environmental conditions that influence the circadian period. An almost identical process relies on the ortholog proteins CLK-BMAL in mammals. Recent findings have challenged the transcription-translation feedback model and suggest that circadian transcription is an output process and that the post-translational modification of clock proteins is the real central pacemaker mechanism. In the present study, we have manipulated the levels and strength of the CLK-CYC complex. The results demonstrate that its activity is vital for proper period determination and thus indicate that the transcriptional feedback loop is part of the core circadian mechanism.

## Abstract 3

Organisms keep circadian rhythms and use interlocked transcriptional-translational feedback loops as part of the mechanism. This study highlights the importance of transcription for timekeeping.

## Introduction

Circadian rhythms are widespread in nature and help to maintain internal temporal order as well as anticipate daily environmental changes [1]. They use self-sustained biochemical oscillators that generate oscillations at the molecular, physiological, and behavioral levels [2,3].

Results over the past 15 years have highlighted the importance of transcription to circadian biology [4]. In eukaryotic systems, a large fraction of mRNAs, perhaps 10% or more, undergoes circadian transcription (e.g., [5,6]). Circadian transcriptional oscillations contribute to myriad physiological and behavioral outputs in diverse tissues of eukaryotic organisms (e.g., [5,7–10]). Recent data from humans, mice, and flies indicate that numerous syndromes and even pathologies result from a disruption of these daily oscillations [11–16].

A conserved heterodimeric transcription factor, constituted by the proteins Clock and BMAL1 (CLK–BMAL) in mammals and Clock and Cycle (CLK–CYC) in flies, sits at the top of the system that generates circadian transcriptional oscillations [17–22]. These complexes direct the transcription of direct target genes, some of which encode repressors of the activity that leads to their transcription. These repressor proteins, chiefly Timeless and Period in flies or Cryptochrome and Period in mammals, accumulate over the course of many hours and ultimately result in the repression of CLK–CYC or CLK–BMAL activity, respectively [23–28]. A complete cycle takes approximately 24 h and is entrained or reset to exactly 24 h by the daily light–dark (LD) cycle.

These transcriptional cycles constitute the core circadian transcriptional feedback loop of flies and mammals. There are also subsidiary loops involving additional repressors and activators, but genetic evidence indicates that they are less important to circadian timekeeping [29–31]. The circadian transcriptional feedback loop was originally proposed in flies and based on the circadian oscillation of *per* transcription as well as the role of PER in the parallel timing of behavioral and transcriptional oscillations [23,25]. Subsequent evidence made a direct role of PER in transcriptional repression more likely [18,24,32–34].

There is also an important contribution of post-transcriptional and post-translational regulation to circadian timekeeping in both the fly and the mammalian systems. In Drosophila, genetic evidence indicates that major alterations in circadian period result from mutations of key kinase genes, and there is similar evidence in mammals. For example, the key Drosophila clock gene *doubletime* (*dbt*) encodes CKIɛ, and its mammalian relative is also a clock gene [35–37]. This importance of phosphorylation to circadian timekeeping even derives from studies of humans with advanced sleep phase syndrome [11–13,38]. Manipulation of phosphatase activities within Drosophila clock cells also affects circadian period [39,40].

Major targets of these post-translational modifications appear to be the transcriptional repressors PER and TIM. Their modification status as well as the rates with which these modifications take place have a major influence on their degradation rate [35,38,41–50]. Modification of PER may additionally influence its transcriptional repressor activity or the timing of this activity [34,38,51–53]. It is also likely that the repression of CLK–CYC activity occurs at least in part via CLK phosphorylation, which may be mediated by a PER–DBT complex and/or a PER–TIM–DBT complex [42–44,54].

The importance of post-translational modification to period determination has been strengthened by recent results from cyanobacteria [55]. The three key clock proteins—KaiA, KaiB, and KaiC—are transcription factors. However, recombinant versions of these proteins undergo circadian oscillations of association and modification state in vitro (KaiC has autokinase and autophosphatase activity) in the absence of transcription and without nucleic acids [56,57]. These results make it very likely that the core circadian system in cyanobacteria is predominantly if not exclusively post-translational and suggest that circadian transcriptional regulation is a downstream output feature, unnecessary for core circadian timekeeping. This raises the possibility that a similar situation occurs in flies and mammals: the core circadian system may be primarily post-translational (e.g., based on the temporal modification of PER and TIM). Consistent with this notion, Yang and Sehgal have shown that circadian locomotor activity rhythms can occur with *per*- and *tim*-expressing transgenes missing their natural promoters [58]. This work extended previous indications that behavioral rhythms require PER activity but do not require circadian transcription of the *per* gene [59].

To pursue the contribution of transcription to core circadian timekeeping in Drosophila, we have analyzed the in vivo effects of a CYC–viral protein 16 (VP16) fusion gene. VP16 is a potent transcriptional activator derived from Herpes virus [60] and imparts to the CLK–CYC-VP16 complex enhanced transcriptional activity relative to the normal CLK–CYC heterodimeric complex. This is based on activity in S2 cells as well as flies expressing CYC-VP16. These flies also have increased levels of CLK–CYC direct target gene mRNAs, including those from *per* and *tim*. Moreover, the CYC-VP16-expressing flies have short periods, implicating circadian transcription in period determination. Taken together with more detailed molecular analyses of these flies as well as behavioral assays of strains missing the normal *per* promoter, we suggest that CLK–CYC-mediated transcription of the *per* gene is important for period determination.

## Results

**Figure 1:**
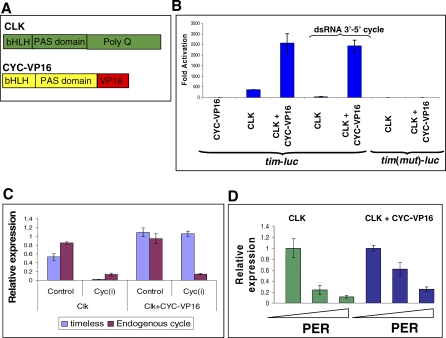
CYC-VP16 Increases the Transcriptional Strength of CLK–CYC Heterodimer (A) Schematic diagram of the CLK protein (top) and of the CYC-VP16 hybrid protein (bottom) showing the protein domains of those molecules. (B) Effect of *cyc*-*vp16* expression (100 ng of *cyc*-*vp16*-expressing plasmid, *pAc*-*cyc*-*vp16*) and/or *Clk* expression (10 ng of *pAc*-*Clk*) on the transcription of *tim-luc* (first five bars) or *tim*(*mut*)-*luc* (last two bars) in S2 cells. Some cells were treated with dsRNA against the 5′ and 3′ UTR regions of the *cyc* gene as described in the Materials and Methods section (fourth and fifth bars). In all cases, cotransfection with *pCopia-Renilla luciferase* was performed to normalize for cell number, transfection efficiency, and general effects on transcription. For each condition a normalized firefly/Renilla luciferase value was obtained by setting the ratio without any addition of *cyc-vp16* or *clk* to 1. A representative experiment is shown. For each condition two experiments with duplicates were performed. Error bars represent standard error of the mean. (C) Similar experiment to the one performed in (B) but assessing the levels of *tim* and *cyc* mRNAs by real-time PCR. Control or *cyc*(i) refers to dsRNAs against *green fluorescent protein* or untranslated regions of *cyc*. Expression values are reported as a ratio of *tim* or *cyc* over control (*rp49*). The experiment was performed twice, and the results were averaged. The error bars indicate standard error of the mean. (D) PER repression of CLK-mediated transcription in presence (100 ng of *pAc*-*cyc*-*vp16*) or absence of *cyc*-*vp16*; 0, 50. or 150 ng of *pAc*-*per* were used. For each condition a normalized firefly/Renilla luciferase value was obtained by setting the ratio with the addition of *pActin*-*Clk* to 1. A representative experiment is shown. Two experiments with duplicates for each condition were performed.

To manipulate the transcriptional activation potential of the CLK–CYC heterodimer, we generated a fusion protein between the CYC protein and the strong and well-characterized viral transcriptional activator VP16 (Figure 1A) [60]. Current indications are that all activator activity of the CLK–CYC heterodimer normally comes from the polyglutamine region of CLK (Figure 1A) [18], so we considered that VP16 might increase the activity of a CLK–CYC-VP16 heterodimer. As an initial assay, DNA encoding the fusion protein was transfected into S2 cells along with a standard *timeless* promoter-*luciferase* (*tim*-*luc*) reporter gene [18,61], which responds well to CLK–CYC activity.

Transfection of the fusion protein gene has little or no activity (Figure 1B). This is expected and reflects the absence of its partner CLK from S2 cells [18]. In contrast, transfection of a CLK gene alone partners with endogenous CYC and potently increases reporter gene activity (Figure 1B), identically to what has been reported previously [18]. Cotransfection of CLK with CYC-VP16 increases activity a further 5-fold (Figure 1B), which presumably reflects the transcriptional activation potential of VP16. Importantly, cotransfection of CLK with CYC or with another VP16 fusion protein (GAL4-VP16) has no effect over transfection with CLK alone (Figure S1A and unpublished data). An assay of endogenous *tim* mRNA expression by real-time PCR and TIM protein by western blotting gives rise to similar results: CYC-VP16 alone has no activity, whereas CLK plus CYC-VP16 cotransfection has considerably more activity than CLK alone (Figure 1C and Figure S1B). Moreover, coexpression of CYC-VP16 rescues activity of the truncated CLK^Jrk^ protein in this tissue culture assay system (Figure S1C); CLK^Jrk^ is missing most of its activation domain [17].

CLK-driven transcription is inhibited by double-stranded RNAs (dsRNAs) against the 5′ and 3′ untranslated regions (UTRs) of the endogenous *cyc* mRNA present in S2 cells (Figure 1B and 1C). In contrast, activity due to cotransfection of CLK and CYC-VP16 is insensitive to incubation with the same dsRNAs (Figure 1B and 1C). This is because the CYC-VP16 expression plasmid does not carry the *cyc* UTRs. The result indicates that most CLK activity is derived from the CLK–CYC-VP16 heterodimer. Neither CLK–CYC nor CLK–CYC-VP16 has activity on a *tim*-*luc* reporter with mutant E-boxes [61], indicating that the CLK–CYC-VP16 fusion has DNA-binding properties similar to wild-type CLK–CYC (Figure 1B). Because there is no detectable endogenous *per* expression in S2 cells, even after *clk* expression [62], the higher target gene mRNA levels are likely the consequence of a stronger transcriptional activation independent of any possible weaker PER-mediated repression on CYC-VP16.

Cotransfection with *per* cDNA inhibits CLK–CYC-VP16 activity, similar to what is observed for CLK–CYC activity (Figure 1D) [18,34] Given the entirely different nature of the VP16 activator compared to the polyglutamine region of CLK and the 5-fold increase in activity, this suggests that *per* repression involves a similar inhibition of CLK–CYC and CLK–CYC-VP16, probably an inhibition of DNA binding [54]. The similar properties of the two heterodimers are despite the much more potent activity of the former.

**Figure 2:**
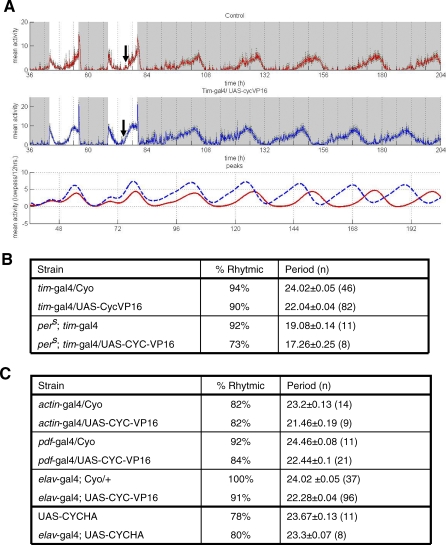
Expression of *cyc*-*vp16* in Pacemaker Neurons Shortens the Period of Behavioral Rhythms (A) Comparison of circadian locomotor behavior of control flies (*uas*-*cyc*-*vp16*/+, top tracing) and flies expressing *cyc*-*vp16* under the control of the *tim* driver (*tim*-*gal4*/*uas*-*cyc*-*vp16*, middle tracing). In each case, the behavior is shown in average actograms. The arrow indicates the phase of evening anticipation for each fly strain. The light timing is indicated by alternating white and gray background areas, with white representing the illuminated interval of the LD condition (ZT0–12) and gray representing the dark period (ZT12–24 and DD period). To facilitate the identification of peaks in the control and *tim*-*cyc*-*vp16* datasets the data were smoothed with a low-pass filter set with a cutoff of 12 h (bottom tracing). (B) Period length of fly strains overexpressing *uas*-*cyc*-*vp16* using the *tim*-*gal4* driver and control flies. (C) Behavioral analysis of fly strains expressing the *uas*-*cyc*-*vp16* or *uas*-*cyc* transgenes in combination with different *gal4* drivers.

To generate flies with *cyc*-*vp16* expression in circadian cells, we created *uas*-*cyc*-*vp16* transgenic flies and crossed them to *tim*-*gal4* driver lines. We then assayed circadian locomotor behavior in these *tim*-*cyc*-*vp16* flies (Figure 2A and 2B, top). They were robustly rhythmic with ∼22-h periods, approximately 2 h shorter than those of wild-type flies. Figure 2C summarizes comparable period shortening by *uas*-*cyc*-*vp16* combined with a highly spatially restricted circadian driver (*pdf*-*gal4*) and with two broader expression drivers (*actin*-*gal4* and the pan-neuronal *elav*-*gal4*). This indicates that the ∼22-h period is not an idiosyncrasy of the *tim*-*gal4* driver. Moreover, the short period was not simply caused by *cyc* overexpression. This is because *elav*-*cyc* flies (*uas*-*cyc* rather than *uas*-*cyc*-*vp16* in combination with the same *elav*-*gal4* driver) have a wild-type–like period (Figure 2C, bottom). We thus attribute the period-shortening effect to increased transcriptional activity from the CLK–CYC-VP16 heterodimer within circadian cells.

Consistent with this interpretation is the period of *tim*-*cyc*-*vp16* in combination with the classic *per^s^* allele; these flies have ∼17-h periods, 2 h shorter than the canonical *per^s^* 19–20 h phenotype (Figure 2B, bottom). The additive nature of *tim*-*cyc*-*vp16* and *per^s^* suggests that they shorten period in independent ways, the former by increasing transcription of CLK–CYC direct target genes and the latter by causing more rapid PER turnover [50].

To further study the period-shortening effect of *tim*-*cyc*-*vp16*, we characterized the molecular clock of these flies. To this end, we added a *tim*-*luc* or a *per*-*luc* reporter gene to the *tim*-*cyc*-*vp16* strain (generating *tim*-*luc*-*cyc*-*vp16* flies or *per*-*luc*-*cyc*-*vp16* flies).

**Figure 3:**
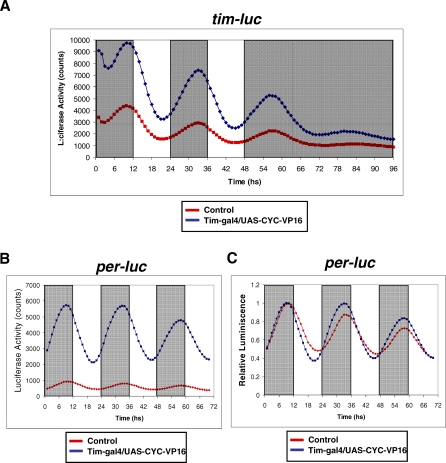
Expression of *cyc*-*vp16* Affects CLK-Mediated Transcription in Cultured Fly Wings (A) Luciferase recordings from control (*uas*-*cyc*-*vp16*/+) and *tim*-*cyc*-*vp16* flies using the *tim*-*luc* reporter. Light timing is indicated by alternating white and gray background areas, with white representing the illuminated interval of LD (ZT0–12) and gray representing the dark period (ZT12–24). After 3 d in LD conditions the assay was conducted in DD conitions. The results are the average of ten (*tim*-*cyc*-*vp16*) and 13 (control) pairs of fly wings. (B) Luciferase recordings from control (*uas*-*cyc*-*vp16*/+) and *tim*-*cyc*-*vp16* flies utilizing the *per*-*luc* reporter. The results are the average of 24 pairs of fly wings of each genotype. (C) Both curves in (B) were normalized to their maximum and then plotted together.

The expression of *luciferase* is robustly rhythmic in *tim*-*luc*-*cyc*-*vp16* flies and isolated wings. The patterns are similar to those of wild-type *tim*-*luc* flies, but luciferase levels were about 2–3 times higher (Figure 3A for isolated wings and Figure S2A for intact flies). This is a comparable activity difference to what was observed above between CLK–CYC and CLK–CYC-VP16 in S2 cells (Figure 1B and 1C). Robust cycling and an even greater activity difference are observed with the *per*-*luc* reporter gene (Figure 3B).

Normalization to the first peak of the oscillations in Figure 3B and 3A allowed a useful comparison between the controls and the *per*-*luc*-*cyc*-*vp16* and the *tim*-*luc*-*cyc*-*vp16* profiles (Figure 3C and Figure S2B). The normalized pairs are very similar, but the CYC-VP16 curves are phase-advanced as they decrease more rapidly and then increase more rapidly during the next cycle (Figure 3C and Figure S2B). The peaks remain coincident, almost certainly reflecting entrainment to the superimposed 24-h LD cycle. Careful observation of the *tim*-*luc* reporter in constant darkness (DD) conditions reveals shorter circadian period in CYC-VP16 flies, in parallel with the behavior (Figure S2B). The damping oscillations of the wing transcriptional reporters in DD (always true in our hands) precluded a precise period determination.

**Figure 4:**
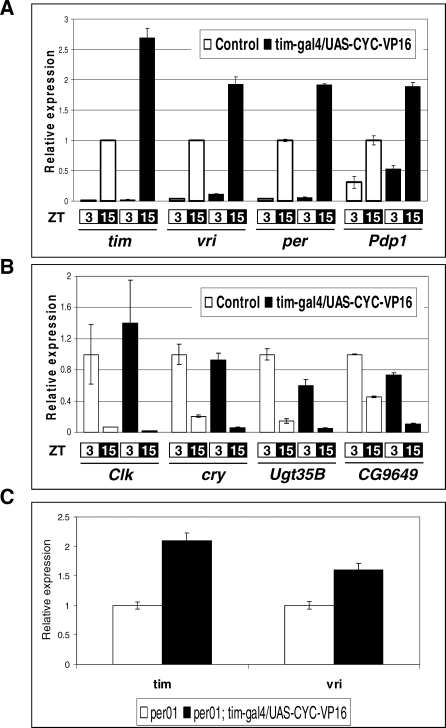
mRNA Levels of Direct CLK Targets Are Selectively Affected in *tim*-*cyc*-*vp16* Flies (A) mRNA expression value for control (*uas*-*cyc*-*vp16*/+, white) and *tim*-*cyc*-*vp16* flies (black) for two timepoints (ZT3 and ZT15) of four direct *Clk* targets: *tim*, *vri*, *per*, and *pdp1*. The data were obtained by microarray (n = 2 for each genotype and timepoint). The data were normalized to the maximum value obtained in the control flies. Error bars indicate standard error of the mean. (B) mRNA expression value for control (*uas*-*cyc*-*vp16*/+, red) and *tim*-*cyc*-*vp16* flies (blue) for two timepoints (ZT3 and ZT15) of four genes (*Clk*, *cry*, *Ugt35B*, and *CG9649*) that are not direct CLK targets and that oscillate in control flies with opposite phase than the genes shown in (A). The data were obtained by microarray (n = 2 for each genotype and timepoint). The data were normalized to the maximum value obtained in the control flies. Error bars indicate standard error of the mean. (C) Effect of *cyc*-*vp16* in *per^01^* mutants flies measured by quantitative PCR. Un-entrained *per^01^* or *per^01^*; *tim*-*gal4*; *uas*-*cyc*-*vp16* flies were harvested. RNA was extracted from fly heads, and quantitative PCR was performed. Expression values for each transcript and timepoint were generated by dividing the *vri* or *tim* mRNA signal by the expression value for a control non-circadian mRNA (*rp49*). Expression values are reported as a ratio of *tim* or *vri* over *rp49* expression. We assigned a value of 1 to the ratio obtained for control flies and proceed as in (A). The data are the average of the normalized *vri* or *tim* expression values for three independent RNA samples. The error bars indicate standard error of the mean.

To compare these reporter effects with those on bona-fide circadian mRNAs, microarray assays were performed on *tim*-*cyc*-*vp16* head RNA from Zeitgeber time 15 (ZT15) and ZT3 (the timepoints when the CLK target genes have the peak and trough mRNA amounts in wild-type flies) and compared to the same timepoints from wild-type flies (Figure 4A and 4B). CLK–CYC direct target gene (*tim*, *per*, *vrille* (*vri*), and *par domaine protein 1* (*pdp1*)) mRNA peak levels increase 2–3-fold, and an increase is also observed in trough levels (Figure 4A). The increase in trough levels suggests that they normally result from residual CLK–CYC activity that resists repression and/or that there is a minority of CLK–CYC-expressing cells that lack a robust circadian repression system. The microarray results are qualitatively similar although quantitatively less striking than the reporter gene assays shown above (Figure 3A and 3B). This may reflect the longer half-lives of the CLK–CYC direct target gene mRNAs relative to the *luciferase* reporter mRNAs or another level of post-transcriptional regulation. It is also possible that the reporter genes have a larger transcriptional response to CYC-VP16 than the CLK–CYC direct target genes.

In contrast to these direct target genes, maximal values for cycling mRNAs that peak at the opposite time of day are not increased in *tim*-*cyc*-*vp16* flies (Figure 4B). Trough levels are decreased, however, suggesting that this might reflect an increase in the level of a transcriptional repressor protein, itself the product of a CLK–CYC direct target gene (e.g., VRI [29,30]).

We also tested whether the increase in CLK-mediated transcription was predominantly due to impaired *per* repression. To this end, we measured the effect of the CYC-VP16 protein in a *per* null mutant (*per^01^*) background [63]. The *tim* and *vri* mRNA levels are increased in *per^01^* flies, comparable to the increase in the S2 cell (also without PER) experiments (Figure 1B). This indicates that transcription is increased independent of any more subtle effects on *per* repression.

Although we attribute the shorter period of the *cyc*-*vp16* flies to a direct enhancement of transcription, it is still possible that the VP16 activation domain has a subtle effect on some other aspect of repression, which then only indirectly enhances transcription. Therefore we decided to assay the periods of transgenic flies carrying increasing numbers of copies of the *Clk* genomic region. Introduction of additional copies of the *Clk* transgene shortens circadian period and increases CLK–CYC-mediated transcription similar to the effects of the *cyc*-*vp16* transgene (Figure 4A and 4B).

**Figure 6:**
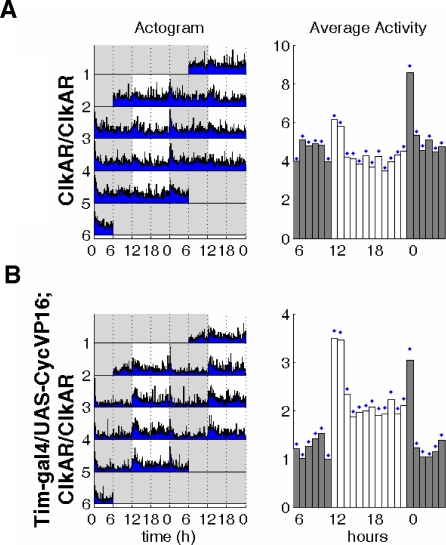
Expression of *cyc*-*vp16* Partially Rescues LD Behavior in *ClkAR* Flies (A) Locomotor behavior of *ClkAR* mutant flies in LD conditions. Four standard days are shown, with timing indicated by alternating white and gray background areas with white representing the illuminated interval of LD (ZT0-12) and gray representing the dark period (ZT12-24). The behavior is shown in actograms (left) and averaged actograms (right). (B) Same as in (A), but using *tim*-*gal4*/*uas*-*cyc*-*vp16*; *ClkAR*/*ClkAR* flies.

Homozygous *ClkAR* flies have significantly diminished levels of functional CLK and very low amplitude transcriptional oscillations of core clock genes [64]. As a consequence, these mutant flies do not have circadian activity patterns in DD or even in standard LD conditions. In addition they do not show the typical burst of activity at the beginning of the light cycle present in wild-type flies (lights-on startle response). Because CYC-VP16 increases CLK-driven transcription, we tested it for rescue of circadian activity in the *ClkAR* mutant background. Although introduction of CYC-VP16 into the *ClkAR* background failed to rescue circadian locomotor activity rhythms in DD conditions, most of the abnormal features of LD behavior conditions were restored: this included the presence of behavioral cycles (higher diurnal than night activity) as well as the lights-on startle response (Figure 6).

**Figure 7:**
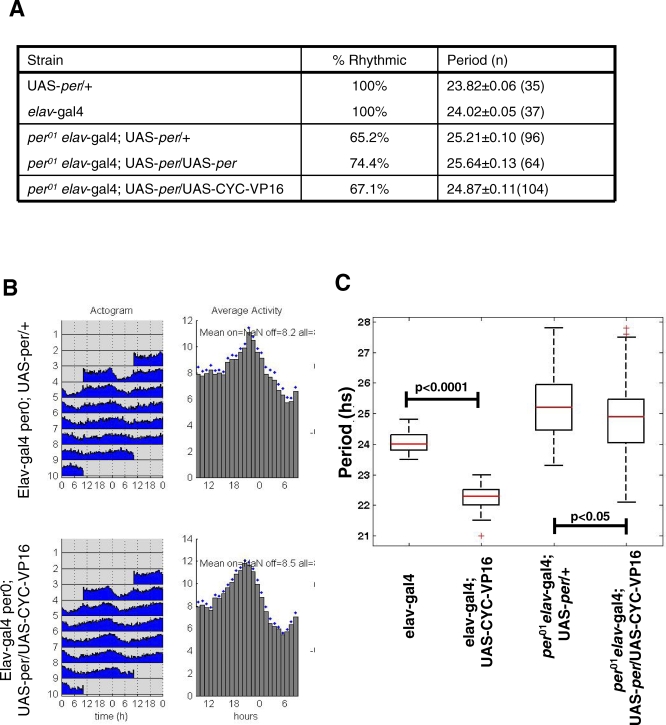
Period Effect of *cyc*-*vp16* Is Mainly Mediated by *per* Transcription (A) Period length analysis of fly strains overexpressing *uas*-*cyc*-*vp16* using the *elav-gal4* driver and control flies in different genetic backgrounds. (B) Locomotor behavior of *per^01^*; *elav-gal4*; *uas*-*per* flies with or without a *uas*-*cyc*-*vp16* transgene in DD conditions. The behavior is shown in actograms (left) and averaged actograms (right). (C) Box plot showing the period distribution of the specified flies. The *p*-values correspond to a *t*-test performed among the indicated samples.

The effect of CYC-VP16 on the transcriptional profiles of the reporters and CLK–CYC direct target mRNAs suggested that the period-shortening effect might be simply due to a CYC-VP16-mediated change in the timing or level of *per* transcription. To test this possibility, we assayed the period of *tim*-*cyc*-*vp16* flies in the context of *uas*-*per* (i.e., a *period* gene that can be driven constitutively by GAL4 but not by CLK–CYC or by CLK–CYC-VP16). Importantly, Sehgal and co-workers [58] have shown previously that *uas*-*per* can rescue the arrhythmic *per^01^* genotype (*per^01^*; *elav*-*gal4*; *uas*-*per*), and we verified this finding (Figure 7A). Importantly, the *elav*-*gal4* driver in combination with *uas*-*cyc*-*vp16* (and a wild-type *per* gene) also manifests the ∼2-h period shortening as shown above (Figure 2C). However, these two transgenes in combination with the *uas*-*per* and *per^01^* only shorten circadian period by 20 min (Figure 7A–7C, and Figure S3B). This indicates that an increase in the levels and/or timing of *per* transcription is a major contributor to CLK–CYC-VP16 period shortening*.* We also note the broad distribution of individual fly periods from genotypes containing the *uas*-*per*; *per^01^* combination compared to the much tighter distribution in genotypes containing a proper *per* promoter (Figure 7C and Figure S3C); this is an additional indication that *per* transcription contributes to period determination (see Discussion section).

This role of *per* transcription is consistent with previous reports showing a relationship between *per* gene dose and behavioral period: more *per* genes cause shorter periods [65–67]. To determine if other ways of increasing *per* transcription also give rise to period shortening, we compared behavioral period between genotypes with one or two doses of *uas*-*per* (Figure 7A and Figure S3C). Rather than shortening period, however, the extra copy of *uas*-*per* slightly lengthens it. This is consistent with previous reports showing that overexpression of a *uas*-*per* transgene does not shorten period [44,58,68]. Taken together with other data shown above, we conclude that the short period of *tim*-*cyc*-*vp16* requires not just increased levels of *per* mRNA but proper timing of the *per* transcriptional increase.

## Discussion

To address the role of transcription in core circadian timekeeping in the Drosophila system, we have analyzed the effects of a *cyc*-*vp16* fusion gene in S2 cells as well as in flies. VP16 is a potent and well-studied transcriptional activator, which imparts to the CLK–CYC-VP16 heterodimer enhanced activity relative to that of the normal CLK–CYC complex. This increased activity is manifested with reporter genes, and transgenic flies also have increased levels of CLK–CYC direct target gene mRNAs, including those from *per* and *tim*. Importantly, the *cyc*-*vp16*-expressing flies have a short period, implicating circadian transcription in period determination. As this short period and proper period control more generally require a *per* promoter, we suggest that CLK–CYC-VP16 drives increased *per* transcription, which leads to more rapid accumulation of PER and a consequent advanced phase of *per* repression. This is also consistent with reporter gene profiles in *cyc*-*vp16*-expressing flies. The results indicate that circadian transcription contributes to core period determination in *Drosophila*.

**Figure 5:**
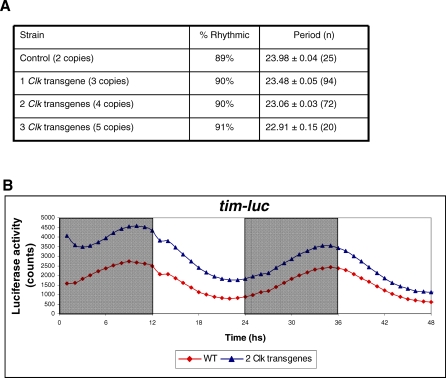
Increase of *Clk* Copy Number Shortens the Circadian Clock (A) Behavioral analysis of fly strains with one, two, or three doses of a *Clk* transgene. Transgenes in the second and third chromosome were used. All of the flies assayed are wild type for the endogenous *Clk* locus. (B) Luciferase recordings from control (yw) and flies carrying two doses of a *Clk* transgene utilizing the *tim*-*luc* reporter. Light timing is indicated by alternating white and gray background areas, with white representing the illuminated interval of the LD condition (ZT0–12) and gray representing the dark period (ZT12–24). The results are the average of 13 (two copies of the *Clk* transgene) and 18 (control) pairs of fly wings.

This conclusion fits with several other pieces of data from the *Drosophila* system. First, recent studies have identified the transcriptional repressor–encoding gene *clockwork orange* (*cwo*) as a clock gene [62,69,70]. The protein product synergizes with PER and aids the repression of CLK–CYC direct target genes. Importantly, mutations in *cwo* or changes in *cwo* expression cause substantial period changes. Second, an increase in *per* gene dose leads to flies with short periods. There is a decrease of approximately 0.5 h for each additional gene copy up to about four copies, which have a ∼22-h period (e.g., [65]). Third, a hemizygous deletion that includes *clock* lengthens circadian period by about 0.5 h [17]. Although this deletion removes more DNA than just *clk* (including the adjacent clock gene *pdp1*), our results indicate that additional copies of the *clk* locus indeed shorten the circadian period of otherwise wild-type flies (Figure 5). All of these observations are qualitatively similar to the increase in transcription and period shortening caused by expression of *cyc*-*vp16* in flies.

Because of the molecular analyses (Figure 3 and Figure S2), we suspect that it is the timing of *per* transcription rather than a simple increase in *per* mRNA levels that causes the period shortening by expression of *cyc*-*vp16*. As the reporter genes contain proper *per* and *tim* promoters, their profiles indicate that *per* and *tim* transcription decreases more steeply and then increases more steeply in the *cyc*-*vp16* flies (Figure 3C and Figure S2B). The steeper decrease presumably reflects a faster accumulation of active PER repressor, and the steeper increase reflects the enhanced potency of CLK–CYC-VP16. In addition, we note that an increase in *per* dose with a *uas*-*per* transgene slightly increases rather than decreases period (Figure 7A and Figure S3C) [44,58]. This genetic requirement for the *per* promoter also emphasizes the contribution of proper transcriptional regulation to period determination.

The increased transcriptional potency of CLK–CYC-VP16 is unlikely to be a consequence of impaired PER-mediated repression, due in turn to some structurally anomalous feature of the artificial fusion protein. This is because the stronger activation of CLK direct targets by the CLK–CYC-VP16 dimer is apparent even in the absence of PER (Figures 1C and 4C). Shorter periods due to more potent transcription is also the conclusion of Figure 5, which shows that increasing *clk* gene dose (an independent and “more natural” way to increase CLK-mediated transcription) leads to molecular and behavioral changes that resemble those observed in *tim*-*cyc*-*vp16* flies. Finally, *cyc*-*vp16* expression rescues several aspects of the *ClkAR* phenotype (Figure 6). This suggests that these features are due to low direct target mRNA levels, which are increased by the more potent CLK–CYC-VP16 complex. The failure to rescue the behavioral arrhythmicity of homozygous *ClkAR* flies may reflect a requirement for minimal CLK levels, which would not be expected to increase by the addition of CYC-VP16.

The robust behavioral and molecular rhythms of *cyc*-*vp16* flies (Figure 2 and 3) more generally indicate that CLK–CYC-VP16 circadian function, including the mechanism(s) that temporally activate or repress transcription of this hyperactive complex, must be similar to those that regulate the activity of the wild-type CLK–CYC complex. This is also because the increased transcription as well as RNA levels in *tim*-*cyc*-*vp16* flies suggests that most CLK–CYC direct target gene transcription is carried out by CYC-VP16 rather than endogenous CYC. Because the VP16 activation domain almost certainly functions differently from the CLK polyglutamine region, this indicates that the recruitment of specific activator and/or repressor proteins is unlikely to play a prominent, mechanistic role in the circadian regulation of transcription. A more likely mechanism involves the cyclical inhibition of CLK–CYC DNA binding. Importantly, this notion is consistent with recent chromatin immunoprecipitation results from the mammalian as well as the fly system [54,71]. Nonetheless, we suggest that *per* transcription as well as DNA binding of the CLK–CYC dimer to *per* E-boxes is the actual timekeeper of the circadian cycle during the mid-late day, when they are both increasing. This predicts that the additional activation power of VP16 indirectly shortens the DNA binding time of the CLK–CYC-VP16 dimer by accelerating the rate of PER accumulation and function. This hypothesis also fits well with the behavioral and molecular defects observed in *cwo* mutant flies [62,69,70].

The emphasis on the *per* promoter is seemingly contradicted by the rhythmicity of flies missing not only this promoter but also the *tim* promoter [58]. In our hands as well, *per^01^*; *elav*-*gal4*; *uas*-*per* flies are largely rhythmic despite weak rhythms, and their average period is near-normal. However, the period distribution of individual flies is unusually broad (Figure 7C and Figure S3C), indicating a contribution of the *per* promoter to the proper control of period within individual flies—even without CYC-VP16. Moreover, luciferase recordings from these transgenic flies show poor or no transcriptional oscillations (unpublished data). These observations suggest that individual neurons from this *per^01^*; *elav-gal4*; *uas*-*per* strain might be impaired even more than indicated by the behavioral rhythms of this strain (i.e., circadian brain circuitry might help to compensate for poor core circadian function within individual cells). This is analogous to the superior circadian performance of behavioral rhythmicity and the suprachiasmatic nucleus (SCN) from mutant mouse strains compared to that of individual tissue culture cells (mouse embryonic fibroblasts) derived from the same strains [72].

The role of circadian transcription described in this study complements the well-documented role of PER, TIM, and CLK post-translational regulation in period determination [34,35,43,44,48,49,54,73–75]. Given the parallel role of mammalian CLK and BMAL1 to CLK and CYC, it would be surprising were there not a similar contribution of circadian transcription to mammals. This suggests that there is a division of labor in animals between transcriptional and post-translational regulation of circadian timekeeping, which may even be temporally segregated. In contrast and as mentioned above, recent indications are that post-translational regulation is the pre-eminent mechanism in cyanobacteria. It is also the case that individual bacterial cells keep excellent circadian time, essentially indistinguishable from the culture [76]. This contrasts with individual eukaryotic cells, for example, separated SCN cells, which show substantially more variation in period than the intact SCN or organism [72,77]. All of these considerations suggest that the intracellular timekeeping mechanism of animals is different from that of cyanobacteria. We suggest that this important difference between systems reflects their separate origins, a view that is supported by the lack of sequence conservation between cyanobacterial and animal clock proteins.

## Materials and Methods

### Plasmids.

*pAc*-*clk*, *pAc*-*per*, Copia Renilla luciferase, and *tim*-*luc* have been described previously [61]. *pAc*-*cyc*-*vp16* was constructed by amplifying the *cyc* coding region and the *vp16* activation domain by PCR and ligating in-frame into *pAcA V5/His6* (Invitrogen). *pAc*-*cyc* was constructed by amplifying the *cyc* coding region and ligating in-frame into *pAcA V5/His6*.

### S2 cell transfection.

S2 cells were maintained in 10% fetal bovine serum (Invitrogen) insect tissue culture medium (HyClone). Cells were seeded in a six-well plate. Transfection was performed at 70–90% confluence according to company recommendations (12 μl of Cellfectin (Invitrogen) and 2 μg of total DNA). In all experiments 50 ng of *pCopia Renilla luciferase* plus 50 ng of the *luciferase* firefly reporter were used. *pBS*-*KS*+ (Stratagene) was used to bring the total amount of DNA to 2 μg.

### dsRNA synthesis and RNAi treatment.

For both procedures we follow the RNAi protocol in S2 cells previously described [34]. Two dsRNAs were synthesized against *cyc*: one containing its 5′ UTR and another containing the 3′ UTR.

### Analysis of gene expression by real-time PCR.

Total RNA was prepared from S2 cells or adult fly heads using Trizol reagent (Invitrogen) according to the manufacturer's protocol. cDNA derived from this RNA (using Invitrogen Superscript II) was utilized as a template for quantitative real-time PCR performed with the Corbett Research Rotor-Gene 3000 real-time cycler. The PCR mixture contained Platinum Taq polymerase (Invitrogen), optimized concentrations of Sybr-green, and the corresponding primers. *tim*: 5′-CCTTTTCGTACACAGATGCC-3′, 5′ –GGTCCGTCTGGTGATCCCAG-3′; *vri*: 5′-GCGCTCGCGATAAGTCTCTA-3′, 5′-CTTTGTTGTGGCTGTTGGTG-3′; *rp49*: 5′-ATCCGCCCAGCATACAG-3′, 5′-TCCGACCAGGTTACAAGAA-3′; and *cyc*: 5′-GGACGAGCGAGATTGACTATA-3′, 5′-TTTGGAGTGTATACAAATGTCG-3′.

Cycling parameters were 95 °C for 3 min, followed by 40 cycles of 95 °C for 30 s, 55 °C for 45 s, and 72 °C for 45 s. Fluorescence intensities were plotted versus the number of cycles by using an algorithm provided by the manufacturer. mRNA levels were quantified using a calibration curve based upon dilution of concentrated cDNA. mRNA values from heads were normalized to that from ribosomal protein 49 (*rp49*).

### Luciferase activity assay.

Forty-eight hours after transfection cells were assayed using the Dual Luciferase Assay Kit (Promega) following the manufacturer's instructions.

### Western blotting.

Lysate for the luciferase activity assay was electrophoresed in 6% SDS-PAGE. The protein was transferred to a membrane. The membrane was blocked and probed with primary and secondary antibodies according to standard techniques. Rat anti-TIM antibody [78] and horseradish-peroxidase-conjugated anti-rat antibody (Sigma) were used.

### Fly strains.

The following drivers were utilized: *tim*-*gal4* [79], *pdf*-*gal4* [80], and *elav*-*gal4* [58]. *per*-*luc*, *tim*-*luc*, *per^s^*, *uas*-cycHA, and *per*-rescued flies (*per^01^ elav*-*gal4*; *uas*-*per*) were previously described [58,63,64,81,82].

### Construction of *uas*-*cyc*-*vp16* transgenic lines.

The *uas*-*cyc*-*vp16* plasmids were generated by cloning a PCR fragment from *pAc*-*cyc*-*vp16* into *pUAST* [83]. This construct was used to generate germ-line transformants by injecting yw; *Ki* *p^p^* *P*[*ry* ^+^Δ*2–3*]/+.

### Construction of *dClk*-V5 14.8 kb transgenic lines.

D. melanogaster RP98-5K6 bacterial artificial chromosome, which contains the complete *dClk* gene, was used as a template (BACPAC Resources Center at Children's Hospital Oakland Research Institute). Four different fragments covering the entire gene were first PCR amplified and cloned into *pBS* vector (first fragment from 7751817 to 7747254 with *Kpn*I and *Sac*I; second fragment from 7747617 to 7745531 with *Kpn*I and *Sac*II; third fragment from 7745570 to 7741748 with *Kpn*I and *Sac*I; fourth fragment from 7741779 to 7736982 with *Xho*I and *Not*I; the position of nucleotides refer to D. melanogaster 3L chromosome sequence). A V5 tag was inserted in the fourth fragment by quick change PCR (Stratagene) in the C terminus just before the stop codon at 7738162. The four fragments were then cut and ligated together in the *pBS* vector using three endogenous restriction sites, *Bgl*II at 7747320, *Nhe*I at 7745537, and *Nco*I at 7741772, resulting in a final *dClk* transgene of 14878 bp (14836 bp of *dClk* and 42 bp of V5 tag) with *Kpn*I on the 5′ and *Not*I on the 3′ ends. The *dClk*-*V5* transgene was then cut and ligated in the *pCaSpeR 4.0* vector, sequenced, and injected into yw embryo (CBRC Transgenic Drosophila Fly Core).

### Locomotor behavior.

Male flies were monitored for 4 d in LD conditions, followed by 4–5 d in DD conditions using Trikinetics Drosophila Activity Monitors. Analyses were performed with a signal-processing toolbox [84]. We utilized autocorrelation and spectral analysis to estimate behavioral cycle durations (periods) and the Rhythm Index to assess rhythm strength [84].

### Real-time monitoring of luciferase activity from whole flies and dissected wings.

Adult male flies and dissected wings were cultured in 12:12 LD conditions, and luciferase was measured as described previously [85]. In the case of the experiments described in Figure 3A and Figure S2B, the assay was performed for three days in LD (12:12 LD) and then in DD conditions.

### Microarrays.

Probe preparation. Total RNA was extracted from fly heads, using Trizol reagent (Invitrogen) according to the manufacturer's protocol. cDNA synthesis was carried out as described in the Expression Analysis Technical Manual (Affymetrix). The cRNA reactions were carried out using the IVT Transcript Labeling Kit (Affymetrix). Affymetrix high-density arrays for D. melanogaster Genome 2.0 were probed, hybridized, stained, and washed according to the manufacturer's protocol.

*Data analysis.* GeneChip.CEL files were analyzed using R (http://www.r-project.org/) and the bioconductor package (gcrma algorithm; http://www.bioconductor.org/). An anti-logarithm (base 2) was applied to the data to obtain the expression values.

## Supporting Information

Figure S1 Specific Effects of CYCVP16 in S2 Cells (A) Expression of *cyc* does not affect CLK-mediated transcription in S2 cells. S2 cells were transfected with 5 ng of *pAc*-*clk*, a *tim*-*luc* reporter and different amounts of *pAc*-*cyc* plasmid. The data analysis was performed as in Figure 1B.
(B) Protein was isolated from nontransfected S2 cells or cells transfected with *pAc*-*clk* (10 ng) and/or *pAc*-*cyc*-*vp16* (100 ng). Western blotting with anti-TIM was performed to determine TIM levels.
(C) S2 cells were transfected with 100 ng of *pAc*-*Clk^Jrk^* or 100 ng of *pAc*-*Clk^Jrk^* plus 100 ng of *pAc*-*cyc*-*vp16* plasmid.
(196 KB PPT)

Figure S2 Luciferase Real-Time Recordings from Control and *tim*-*cyc*-*vp16* Flies (A) Luciferase recordings from whole flies for the different fly strains were performed as described in the Materials and Methods section.
(B) Each of the curves in (A) (fly wing Luciferase recordings) was normalized to its maximum value and then plotted together. In the lower right box, an amplification of the marked region is shown.
(351 KB PPT)

Figure S3 Representation of the Period Spread among Individuals of Different Fly Strains The *y*-axis corresponds to the relative frequency. The dataset is identical to that displayed in Figure 7A.
(166 KB PPT)

### Accession Numbers

Accession numbers for genetic sequences mentioned in this paper from the National Center for Biotechnology Information (http://www.ncbi.nlm.nih.gov) are the D. melanogaster RP98-5K6 bacterial artificial chromosome, which contains the complete *dClk gene*, (AC010042) and the *D. melanogaster* 3L chromosome sequence (NT_037436.2).

## Abbreviations

*clk*: *clock*
*cyc*: *cycle*
*cwo*: *clockwork orange*
*dbt*: *doubletime*
DD: constant darkness
dsRNA: double-stranded RNA
LD: light–dark
*luc*: *luciferase*
*pdp1*: *par domain protein 1*
*per*: *period*
*rp49*: *ribosomal protein 49*
*tim*: *timeless*
VP16: viral protein 16
*vri*: *vrille*
ZT: Zeitgeber time
